# Image analysis and processing methods in verifying the correctness of performing low-invasive esthetic medical procedures

**DOI:** 10.1186/1475-925X-12-51

**Published:** 2013-06-09

**Authors:** Robert Koprowski, Slawomir Wilczyński, Arkadiusz Samojedny, Zygmunt Wróbel, Anna Deda

**Affiliations:** 1 Department of Biomedical Computer Systems, Faculty of Computer Science and Materials Science, University of Silesia, ul.Będzińska 39, 41-200, Sosnowiec, Poland; 2 Department of Cosmetology, Katowice School of Economics, ul. Harcerzy-Wrzesnia 3, 40-659, Katowice, Poland; 3 Woman’s corner, ul. Moniuszki 59, 41-400, Mysłowice, Poland

**Keywords:** Image processing, Esthetic medical procedures, Laser, Fully automatic

## Abstract

**Background:**

Efficacy and safety of various treatments using fractional laser or radiofrequency depend, to a large extent, on precise movement of equipment head across the patient’s skin. In addition, they both depend on uniform distribution of emitted pulses throughout the treated skin area. The pulses should be closely adjacent but they should not overlap. Pulse overlapping results in amplification of irradiation dose and carries the danger of unwanted effects.

**Methods:**

Images obtained in infrared mode (Flir SC5200 thermovision camera equipped with photon detector) were entered into Matlab environment. Thermal changes in the skin were forced by CO_2_RE laser. Proposed image analysis and processing methods enable automatic recognition of CO_2_RE laser sites of action, making possible to assess the correctness of performed cosmetic procedures.

**Results:**

80 images were acquired and analyzed. Regions of interest (ROI) for the entire treatment field were determined automatically. In accordance with the proposed algorithm, laser-irradiated *L*_*i*_ areas (ROI) were determined for the treatment area. On this basis, error values were calculated and expressed as percentage of area not covered by any irradiation dose (δ_*o*_) and as percentage area which received double dose (*δ*_*z*_). The respective values for the analyzed images were *δ*_*o*_=17.87±10.5% and *δ*_*z*_=1.97±1.5%, respectively.

**Conclusions:**

The presented method of verifying the correctness of performing low-invasive esthetic medical (cosmetic) procedures has proved itself numerous times in practice. Advantages of the method include: automatic determination of coverage error values *δ*_*o*_ and *δ*_*z*,_ non-invasive, sterile and remote-controlled thermovisual mode of measurements, and possibility of assessing dynamics of patient’s skin temperature changes.

## Background

Esthetic medicine market is among the most dynamically developing sectors of industry across the world. Especially popular prove treatments that are low-invasive. According to the American Society of Plastic Surgeons, in the USA alone 13.8 million of low-invasive procedures were performed in 2011, at the estimated value of ca. USD 12.2 billion. Considering trends in population demographics in both developed and developing countries, the tendency of low-invasive esthetic medical procedures to grow in popularity will likely become more dynamic. Owing to the new developments in the medical equipment market it has now become possible to obtain satisfactory results of esthetic medicine procedures, together with a relatively low treatment invasiveness which also means shorter recovery time. Such minimum invasiveness, modest intensity of adverse side effects, coupled with satisfactory effects of treatment is possible, however, only after optimizing procedure parameters.

Action of fractional lasers causes focal ablation of epidermis, whereas radiofrequency-based procedures result in local overheating of both epidermis and corium. At tissue level these agents cause remodeling of collagen fibers and stimulate epidermal regeneration [1-7].

Energy of laser radiation or radiofrequency energy is delivered to patient’s skin in the form of pulses which affect a definite tissue area. Efficacy and safety of treatment using a fractional laser or radiofrequency depend, to a great extent, on precise movement of the therapeutic equipment head across the patient’s skin [8]. In addition, they both depend on uniform distribution of emitted pulses throughout the treated skin area. The pulses should be closely adjacent but they should not overlap [8,9]. Pulse overlapping results in dose amplification and carries the danger of undesired effects [7] and [10].

Known methods for monitoring the efficiency and safety of laser esthetic procedures include optoacoustic [11,12] and optodynamic methods [13]. They are based on using high-sensitivity cameras (detectors) that allow imaging fluorescence phenomena in real time, as well as measuring fluorescence intensity while performing concomitant spectral analysis. Use of these methods has its limitations due to, for example, speed of image acquisition or impossibility of treating the patient in a totally uninvasive manner with increased positioning distances.

In the presented study we aimed to verify the correctness of performing laser-mediated esthetical medical procedures. This was achieved based on automatic calculation of the degree of coverage of the treated area by CO_2_RE laser-sent pulses. The study was performed using the proposed method of analyzing images obtained in infrared mode.

## Materials

We analyzed thermovisual image sequences collected from 15 patients using a Flir SC5200 thermal imaging camera equipped with photon detector. In total, 80 images were analyzed (from patients’ right and left cheek, chin, forehead and nose). Thermal changes in human skin were induced using CO_2_RE laser. All patients were adequately prepared prior to the cosmetic procedure and thermovisual measurements. The error of thermovisual measurement method was minimized by taking into account 1) false estimation of the object’s emissivity, 2) radiation originating from the surroundings and reflected from the object, 3) atmospheric attenuation and scattering and own atmospheric emission, 4) changes in emission from camera optical components, 5) errors intrinsic to methodology of adopted measurement course, 6) air current convection, 7) emotional state of patient, 8) patient’s dress, 9) thermal conductivity of limited and diffuse heat sources, 10) skin vascularization, 11) meals eaten by patient within preceding 24 hrs, 12) crossed radiation, 13) patient’s movements prior to and during examination, 14) undisclosed diseases, and 15) faults in the algorithm. The cited measurement errors can be easily diminished or totally eliminated by assuring constant room temperature (no air movement due to drafts or air conditioning, solar irradiation, radiators, etc.) and securing time necessary to acclimatize a patient in such room (typically half an hour). This is an essential requirement for the majority of thermovisual measurememts.

After minimizing errors due to these causes we carried out measurements according to the methodology presented below.

## Method

Infrared images generated by a thermal scanning camera (Flir SC5200) equipped with photon detector were entered into Matlab environment. The camera has indium antimonide (InSb) detector, with 3–5 μm spectral range, 320×256 pixel resolution and 30×30 μm pixel pitch. Thermal changes in patient’s skin were induced by CO_2_RE laser (CO_2_ type – 10600 nm wave-length, pulsed laser beam emission mode, 4.5 J/cm^2^ energy density, 1–150 mJ impulse energy, 16.7 kHz impulse frequency, 20–3000 μs impulse duration, 10 mm^**2**^ maximum scanned area, 120 μm or 150 μm dot size) [6,14-16].

Application of individual irradiation doses and, thus, induction of thermal changes, was performed manually, by sequentially applying the equipment head to patient’s face. The irradiation procedure was performed by an expert physician who attempted to cover as much as feasible of the whole analyzed area [15]. Synchronization of laser triggering and image acquisition had been programmed. Synchronization error due to operating system delay, data transmission timing and number of stills per second did not exceed 0.1 second. The thermal scanning equipment was positioned during the procedure at ca. 30 cm from patient’s face.

### Preprocessing

Input image *L*(m,n) (where m denotes rows and n denotes columns) at 320×256 pixels, following an increase in resolution to *M*×*N*=480×640 pixels, was filtered using a median filter with *h* mask dimensions *M*_*h*_×*N*_*h*_=3×3. Increase of image resolution was achieved using the nearest neighbor method, avoiding thus new pixel values [17-19]. The generated image *L*_*MED*_ was subjected to processing aimed at detecting regions of interest (ROI). Automatic ROI detection denotes a phase in the image analysis and processing, during which assignment of skin area receiving irradiation dose takes place. This method should work correctly in analyzing temperature changes in areas subjected to therapeutic procedure. Due to close relationship between forcing of temperature change and time of skin temperature reaction onset, this process was followed in more detail.

**Figure 1:**
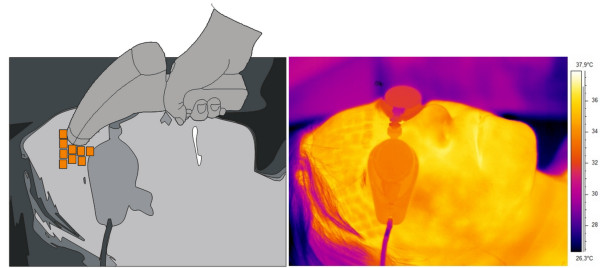
**Pictorial scheme of applying the forcing procedure and placement of thermovision camera with respect to patient’s body (left) and corresponding thermovisual image (right).** The camera is placed 20 cm away from the patient’s skin (in this case from forehead). Squares reminiscent of patient’s forehead areas is another dose of the laser given by an experienced operator. Energy of laser radiation is delivered to patient’s skin in the form of pulses which affect a definite tissue area. Thermal reaction of the skin is strongly related to the kind of forcing, the spot of radiation beam focus on the skin, and the process of human body thermoregulation.

**Figure 2:**
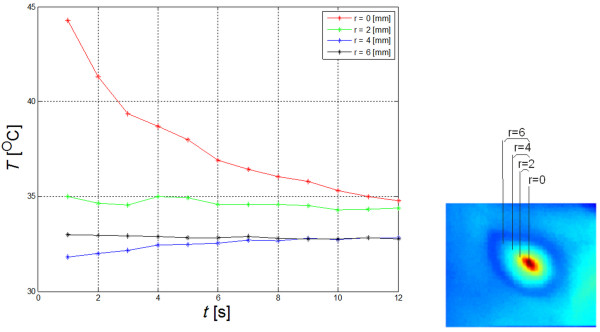
**Patient**’**s skin temperature changes as function of time elapsed.** Point forcing was applied (at *t*=0) in the form of a CO_2_RE laser pulse. Subsequent data points represent average values from the forcing area (2×2 pixels). Figure shows speed of human skin reaction upon pulse forcing – a single microbeam. Laser irradiation creates cone shaped laser channels – the so-called microscopic ablation zones (MAZ). MAZs are lined by a thin layer of coagulated tissue, together constituting the microscopic treatment zones (MTZ). The reaction on laser radiation - MTZ dimensions - differs at various distances *r* from forcing axis center.

**Figure 3:**
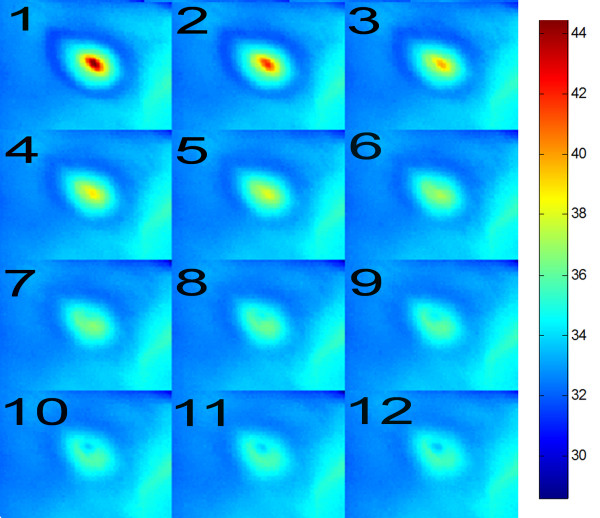
**Sequence of thermovisual images showing temperature changes at 1-sec intervals, reflecting reaction to forcing (CO**_**2 **_**RE laser pulse) in the 2x2 pixel area at the center of the image.** Time measured from initiated forcing ranged from 1 to 12 seconds. During ablative fractional resurfacing treatment, microscopic pieces of skin are vaporized, and a thermal deposit occurs in the dermis. The efficacy and safety of laser treatment is related to the density of superficial microtissue elimination and the thermal deposit left in the dermis. The CO_2_ (CO_2_RE) laser has a high absorption coefficient for tissue water that allows minimal residual thermal damage if the power density is significant enough to cause tissue vaporization that outpaces the speed of thermal diffusion.

**Figure 4:**
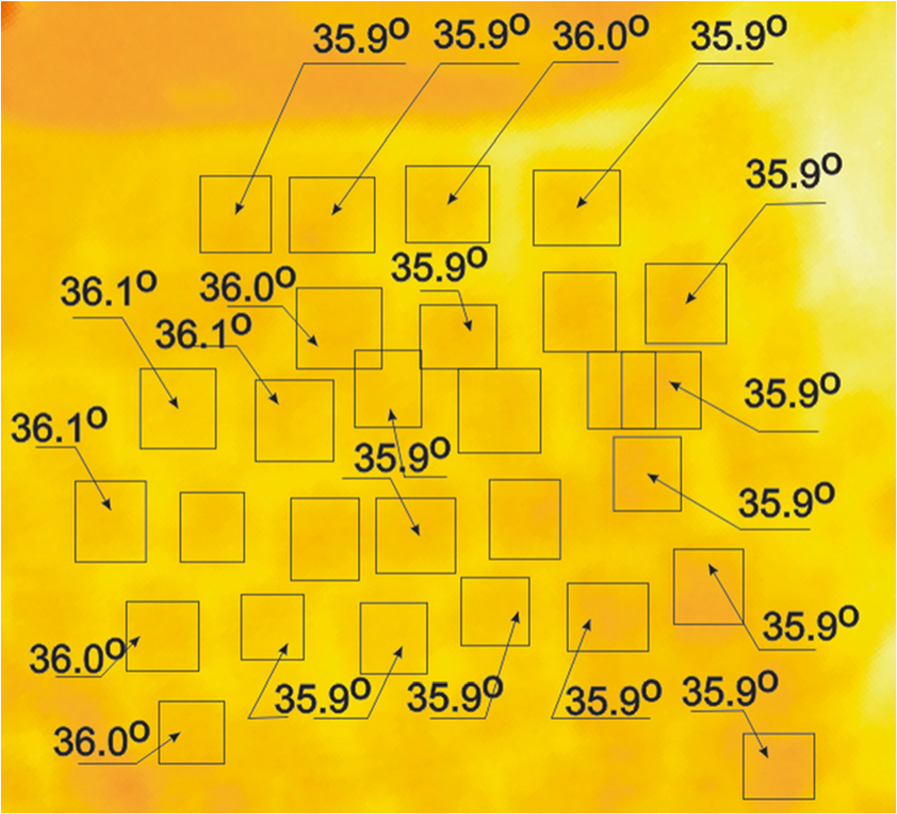
**Temperature distribution (patient’s right cheek) at 2 min after treatment conclusion.** The distribution demonstrates ±0.1°C differences. The areas are not distributed evenly which is the result of imprecise performance of the laser procedure by equipment operator. Figure shows the case of successive overlapping doses of radiation or the formation of untreated spaces. Heat propagation in skin can be modeled with diffusion approximation. With increasing depth and area of thermal injury clinical response increases. Overheating due to an excess number of passes or pulse overlapping can result in scarring.

Thermal reaction of the skin is strongly related to the kind of forcing, the place of radiation beam focusing in the skin, and the process of human body thermoregulation (Figure 1). Inasmuch as radiation beam and surface area it hits can be controlled, the thermoregulation process is an individual human feature. Accordingly, the process of determining ROI had been preceded by assessing human skin reaction speed to set forcing. Figure 2 shows speed of human skin reaction upon pulse forcing. The reaction differs at various distances *r* from forcing axis center. The corresponding images (sequence) are shown in Figure 3. Forcing was defined as single CO_2_RE laser pulse of 1000 μs duration delivered at time *t*=0. Figure 3 shows first measurement after 1 second following forcing and subsequent measurements at 1 second intervals. As seen from the discussed graph the character of changes is typical for first-order inertia object. Steady state appears after a few seconds, however, skin temperature remains elevated by 2.5°C with respect to temperature before treatment. Before the procedure temperature of the involved skin area was 33°C. In all examined patients this state of elevated temperature lasted for no less than 20 minutes. Areas of equipment head application are still visible within that time. They are square areas *L*_*i*_, in agreement with array shape. Based on these experiments it was concluded that for several minutes after procedure conclusion this shape is identical with the area subjected to forcing. The analysis should thus be performed no later than a few minutes after the treatment and no sooner than 10 seconds following the last irradiation dose. By fulfilling these criteria one minimizes a dynamic error linked to human thermoregulation process. Here, it is linked with changes of human skin response to forcing and, more precisely, with duration of CO_2_RE laser treatment in successive spots (Figure 4).

By taking into account these limitations, detection of ROI in the treatment area is unusually simple, due to well visible temperature changes in thermovisual images. Automatic detection of these regions (*L*_*i*_) uses morphological operations with the structural element *SE*. Morphological opening operation *L*_*O*_ causes globalization of image features, in this case ROI.

(1) $$L_{O}=\max_{\mathrm{SE}}\left(\min_{\mathrm{SE}}\left(L_{\mathrm{MED}}\right)\right)$$

It is characterized by highest temperature within the image. Accordingly, the image *L*_*U*_ can be binarized by using a constant threshold *p*_*r*_ that takes into account patient’s skin temperature before the procedure:

(2) $$L_{B}\left(m,n\right)=L_{U}\left(m,n\right)>p_{r}$$

where:

(3) $$L_{U}\left(m,n\right)=\left|L_{O}\left(m,n\right)-L_{\mathrm{MED}}\left(m,n\right)\right|$$

Binarization (*L*_*B*_) may result also in other smaller areas. Removal of smaller, erroneously indicated areas was achieved by labeling procedure (marking of clusters). The largest cluster (area) was then chosen. In all of the acquired thermovisual images this area was indicated correctly. This area (ROI) and *L*_*R*_ image created on its basis were the subject of subsequent analysis.

### Algorithm

**Figure 5:**
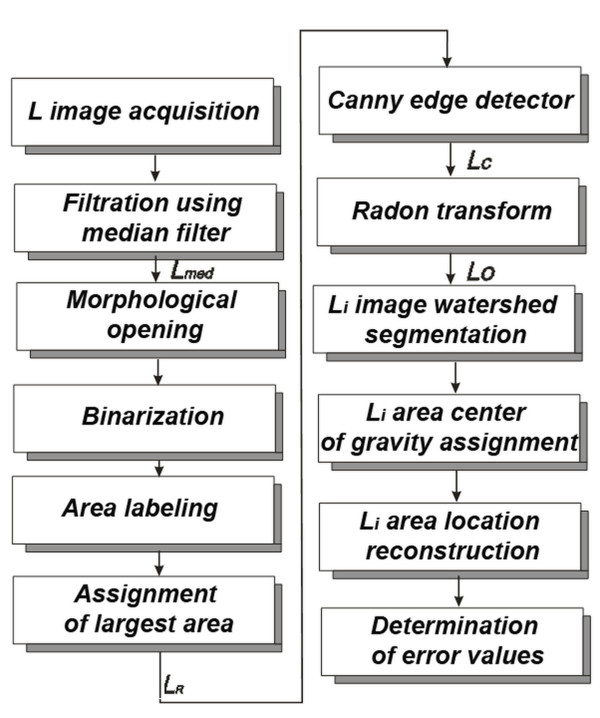
**Flow chart of the two**-**stage algorithm automatically recognizing ROI and *L***_***i ***_**areas.** At the first stage skin area affected by the treatment procedure is segmented (ROI). At the second stage, individual *L*_*i*_ areas are segmented and their position is approximated by a square area. In consequence, percentage values of areas non-covered by laser treatment and areas with overlapping irradiation dose can be calculated.

**Figure 6:**
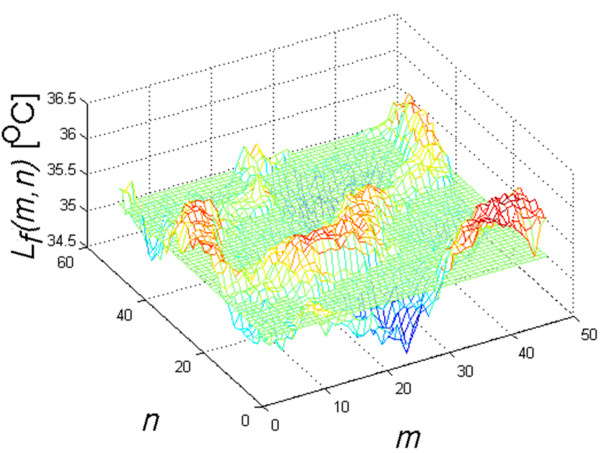
***L***_***f ***_**fragment (input image) temperature changes at 60 s from treatment conclusion.** Apex values represent temperatures higher than those before treatment, while valleys show lower temperatures, respectively. In order to avoid pulse stacking, it is important to know the structure of the beam of a typical CO_2_ laser. Since the distribution of fluences within the laser beam is Gaussian, or bell-shaped, there is a significant difference in ablative depths between the center and the edges of the beam. This may result apparent differences in the distribution of heat on the surface of the skin.

**Figure 7:**
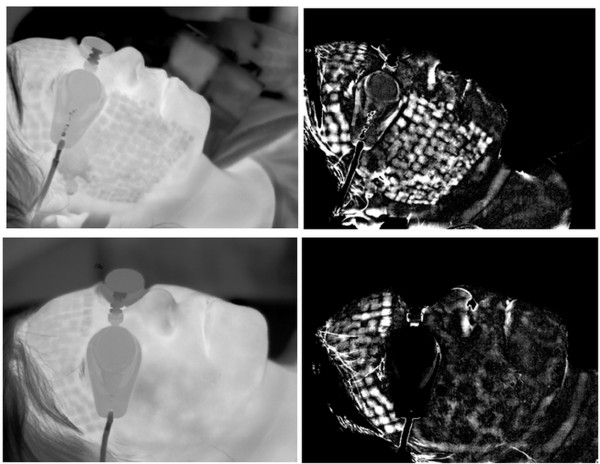
**Intermediate results obtained using the algorithm that automatically recognizes ROI and skin areas subjected to laser intervention.** Right column shows input images *L* while left column shows images that represent the difference of input image *L* and its morphological closing. The left column is thus an intermediate result. At subsequent steps of the algorithm visible clear ROI areas are approximated by square areas resulting from forcing shape. Even at this stage of analysis inaccurate covering of skin surface by laser pulses is seen. Pulse overlapping or stacking may lead to heat accumulation.

**Figure 8:**
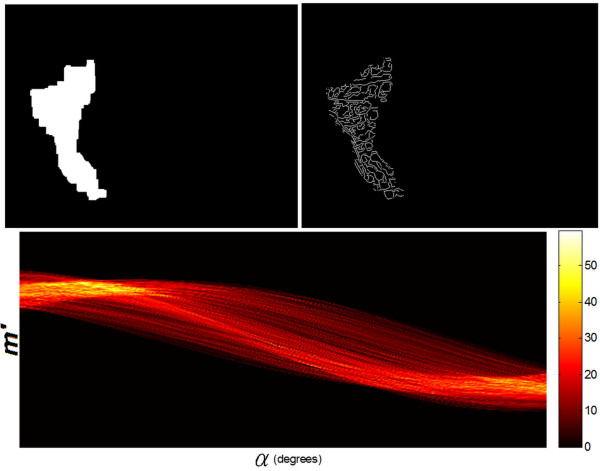
**Final results obtained using the algorithm automatically analyzing unevenness of subsequent irradiations during procedure performance.** Shown are: *L*_*R*_ image with marked ROI (upper left), *Lc* image with edges determined by Canny method (upper right) and the result of Radon transform *L*_*V*_(*n*’,*α*) obtained for *Lc* image (center below).

The block structure of the created algorithm is shown in Figure 5. Input image *L*_*R*_ comprising the measured area was analyzed using Canny edge analysis. As a result, edges of the square areas (*L*_*C*_) of forcing were obtained. Assuming a uniform application of CO_2_RE laser head to the patient’s body by expert equipment operator, *L*_*i*_ areas do not undergo rotation. Accordingly, the next stage of analysis involved detection of angle at which all square areas *L*_*i*_ are found on patient’s body (Figures 6 and 7). To do this, edge images *L*_*C*_ were subjected to operations of Radon transform *L*_*V*_ (Figure 8):

(4) $$L_{V}\left(n^{\prime },\alpha \right)=\sum_{m^{\prime }}L_{C}\left(\mathrm{round}\left(n^{\prime }\cos\left(\alpha \right)-m^{\prime }\sin\left(\alpha \right)\right),\mathrm{round}\left(n^{\prime }\sin\left(\alpha \right)-m^{\prime }\cos\left(\alpha \right)\right)\right)$$

where:

(5) $$\left[\begin{array}{c} n^{\prime }\\ m^{\prime } \end{array}\right]=\left[\begin{array}{cc} \cos\left(\alpha \right) & \sin\left(\alpha \right)\\ -\sin\left(\alpha \right) & \cos\left(\alpha \right) \end{array}\right]\left[\begin{array}{c} n\\ m \end{array}\right]$$

**Figure 9:**
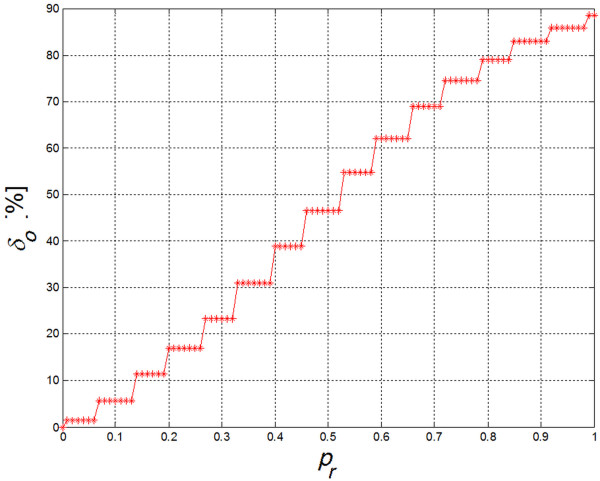
**Changes in *δ***_***o ***_**error values as a function of chosen binarization threshold *p***_***r***_**.**_**.**_ As can be seen, percentage value of free areas is almost linearly dependent on binarization threshold. One cannot conclude unequivocally which binarization threshold will be correct.

**Figure 10:**
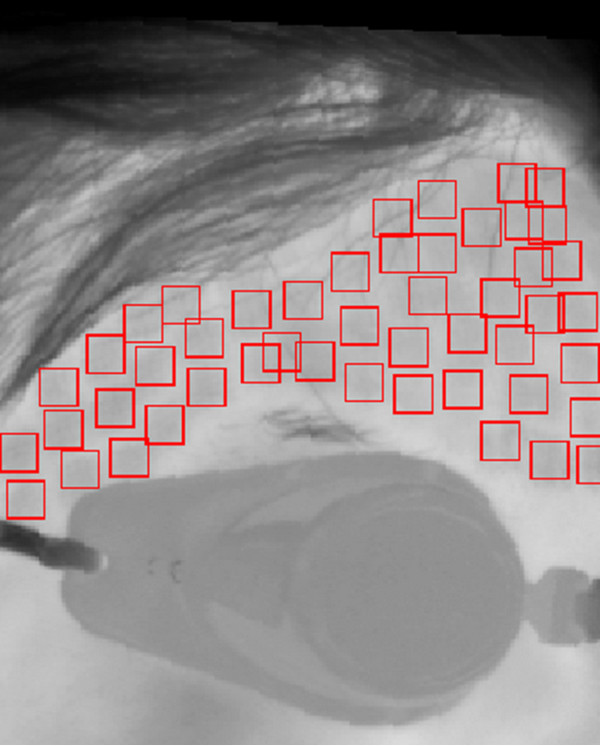
**The result of applying the algorithm.** It automatically recognizes areas subjected to forcing and marks them in red. This makes possible to calculate errors due to the presence of irradiation-overlapping, as well as irradiation-free areas. The evaluation of these areas is critical from a clinical point of view; stacking or overlapping of pulses, and/or excessive number of laser passes all may result in excessive tissue damage. Repeated CO_2_ laser passes will dehydrate and coagulate dermis, which subsequently limits the penetration of laser energy. Because a large part of the heat in subsequent passes or overlapping pulses is not actually used to ablate the skin, the thermal loading of tissue increases.

**Figure 11:**
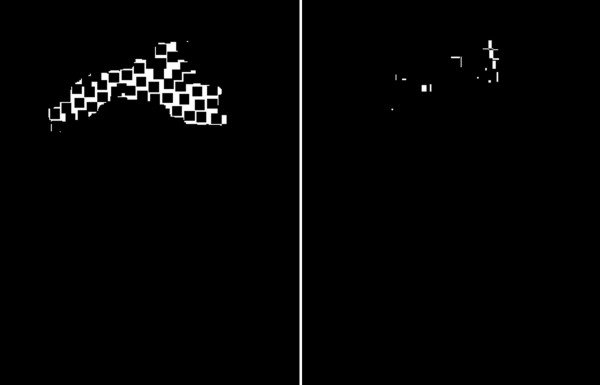
**Image showing irradiation**-**free and irradiation**-**overlapping areas.** These images form the basis for calculating *δ*_*o*_ and *δ*_*z*_ error values. The sum of pixel values seen in the images is calculated with respect to the total sum of pixels in the whole analyzed ROI. Imaging irradiation-free and irradiation-overlapping areas allows to recognize early the critical areas and treat them promptly to avoid permanent sequelae. Although there are individual differences with respect to propensity to side effects, most adverse reactions seen after laser resurfacing appear to be a result of improper treatment technique.

As a result, position of all areas *L*_*i*_ rotated on patient’s skin by angle was equalized. Radon transform can be replaced with Hough transform. At this stage, reconstruction of particular square areas is not essential except for determining one parameter angle α*. The dimensions and location of *L*_*i*_ area is known; only α* angle is unknown. In extreme cases, it is sufficient to determine the maximum from the sum of values for particular rows of the revolving image of *L*_*c*_ edge. Position of *L*_*i*_ areas is detected on *L*_*O*_ image generated in this way. Detection of *L*_*i*_ areas was carried out using watershed segmentation method. Segmentation is implemented on an image subjected to elimination of constant temperature component. Here, binarization with a set threshold did not yield satisfactory results. Figure 9 shows *δ*_*o*_ error value changes as a function of binarization threshold *p*_*r*_ chosen [20]. As seen from the graph, the percentage value of irradiation-free areas is almost linearly dependent on binarization threshold [20-23]. It is not thus possible to state unequivocally which among the chosen binarization thresholds is correct. Following initial segmentation, each area has an assigned center of gravity and is approximated by a rectangular area. Some examples of the results obtained are shown in Figure 10. The process thus consists of separately recognizing each *L*_*i*_ area [24], [25]. As authors’ goal was to evaluate the correctness of the examined procedures, only percentage values of surface areas not receiving treatment and areas with overlapping treatment were calculated. For gap areas lying in between *L*_*i*_ areas percentage values of *δ*_*o*_ error were calculated as a total number of pixels in *L*_*R*_ areas subjected to forcing, with respect to the total number of pixels in uncovered areas (Figure 11):

(6) $$\delta_{O}=\frac{\sum_{m}\sum_{n}L_{R}\left(m,n\right)-\sum_{i}\sum_{m}\sum_{n}L_{i}\left(m,n\right)-\sum_{m}\sum_{n}L_{Z}\left(m,n\right)}{\sum_{m}\sum_{n}L_{R}\left(m,n\right)}\cdot 100\%$$

where *L*_*z*_ is a matrix containing “1” in places where *L*_*i*_ areas superimpose and ”0” in the remaining places.

*δ*_*z*_ error was defined as a percentage value of overlapping areas with respect to the whole *L*_*R*_ area (Figure 11):

(7) $$\delta_{Z}=\frac{\sum_{m}\sum_{n}L_{Z}\left(m,n\right)}{\sum_{m}\sum_{n}L_{Z}\left(m,n\right)}\cdot 100\%$$

Interpretation of errors *δ*_*o*_ and *δ*_*z*_ defined in this way is straightforward. It defines correctness of the performed procedure (forcing). Increased value of *δ*_*o*_ means that the operator did not drive the laser head uniformly, leaving untreated areas. This is not harmful to the patient but requires additional corrective treatment. On the other hand, increased values of *δ*_*z*_ error indicate harm, as patient receives in these areas a double dose of irradiation.

Practical use of this algorithm is demonstrated in the next section.

## Results

**Figure 12:**
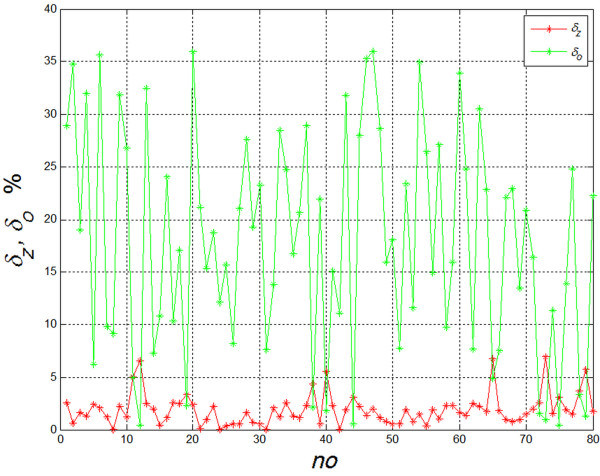
**Changes in *δ***_***o***_**and *δ***_***z ***_**error values for successive cases analyzed.** It can be seen that substantially more errors are due to incomplete laser treatment area coverage than to overlapping coverage.

ROI for the whole treatment region were determined automatically for all of the 80 analyzed images. Next, in accordance with the algorithm given, values of *δ*_*o*_ and *δ*_*z*_ errors were calculated. The obtained results are shown in Figure 12. For low values of *δ*_*o*_ the graph confirms the correlation between values of *δ*_*o*_ and *δ*_*z*_ errors. This means that the operator attempting to fill out the whole treated area causes overlapping of irradiation doses. Mean and standard deviation of the mean for individual errors is *δ*_*o*_=17.87±10.5% and *δ*_*z*_=1.97±1.5%. The error committed by an expert operator pertains primarily to omission of small areas (large value of *δ*_*o*_ error). Errors resulting from overlapping of adjacent *L*_*i*_ areas are small, with single-digit percentage values.

Determination of *δ*_*o*_ and *δ*_*z*_ errors is affected also by other elements specific for the algorithm itself or/and for procedure methodology. These elements include: 1) error due to camera placement with respect to patient’s skin, 2) error due to non-perpendicular placement of laser head with respect to patient’s skin during the procedure of applying irradiation dose, 3) local disturbances in skin thermoregulation, 4) presence of perspiration, 5) interference such as, e. g., patient’s hair falling accidentally onto forehead during the procedure.

In practice, the last two elements predominate: one is caused by skin reaction to temperature and the other by improperly secured hair.

The obtained results as well as *δ*_*o*_ and *δ*_*z*_ error values are affected by personal habits of the operator (technician). It has been noticed that these error values strongly depend on individual habits of the technician and, only to a lesser degree, on the shape of facial area subjected to treatment. Differences in *δ*_*o*_ and *δ*_*z*_ error values for two technicians may vary in a broad range. Due to this reason an automated system of laser triggering has been proposed. The system is based on tracking in visible light the skin areas subjected to treatment (a CCD camera is placed in the laser head). Treated skin areas are memorized using visible light. Following manual relocation (in any direction) of the laser head by a fixed distance, the laser is automatically triggered. Such a system allows minimizing values of *δ*_*o*_ and *δ*_*z*_ errors. In addition, these errors stay independent of the individual habits of an operator. The system is patent-protected [26] and shall be described in detail in future papers.

### Comparison of results with other methods

Contemporary generation of lasers do not offer yet a qualitative analysis of procedures performed using them. It is assumed that an expert laser operator performs the cosmetic procedure correctly, without causing overlapping of irradiation doses. Various practical methods allowing laser beam control and visible image analysis have been reported to date, especially in patent claim literature [27]. Also known have been descriptions of visualization methods using visible light and accomplished by various types of cameras placed, e. g., in laser head [28]. None of these solutions offers, however, analysis of the correctness of procedures performed with laser equipment. Neither temperature fields nor their degree of homogeneity have been assessed. In only a few reports temperature fields and their distribution within the skin were analyzed e. g. [29-31]. In the model proposed by Frahm et al. [29], model simulations of superficial temperature correlated with the measured skin surface temperature (*ρ*>0.90, *p*<0.001). Reported were studies comparing three Infrared Thermal Detection Systems. In this case correlations between ITDS and oral temperatures were similar for OptoTherm (*ρ*=0.43) and FLIR (*ρ*=0.42), but significantly lower for Wahl (*ρ*=0.14, *p*<0.001). Among numerous references pertaining to application of thermovision in medicine only a few e. g. [30-38] have dealt with quantitative (not qualitative) measurements. As an example, Bagavathiappan et al. [33] reported a temperature difference of 0.7–1°C as statistically significant. Based on this one can conclude that thermovisual analysis of human skin does require taking into account numerous factors which interfere with measurement. In the case of the algorithm presented herein only a minute skin fragment is analyzed. An expert laser operator has full control over this fragment, and is capable of minimizing the effect of additional factors to a negligible level.

## Conclusions

The presented method of verifying the correctness of performing laser-mediated esthetic medical procedures has repeatedly proven itself in practice. Its advantages include: 1) automatic determination of *δ*_*o*_ and *δ*_*z*_ error values, 2) non-invasive sterile and remote-controlled thermovisual measurements, 3) possibility of learning how to assess procedure correctness through training, 4) assessment of dynamics of patient’s skin temperature changes, and 5) assessment of correct choice of irradiation dose, treatment length and individual equipment setting.

The described method has been currently used in esthetic medical procedures performed at the Silesian Medical College in Katowice, Poland.

## Competing interests

The authors declare that they have no competing interests.

## Authors' contributions

RK and SW suggested the algorithm for image analysis and processing, implemented it and analyzed 80 images. ZW, AD and AS evaluated the obtained results. All authors have read and approved the final manuscript.
